# Molecular characterization of *Treponema pallidum* subsp. *pallidum* in Switzerland and France with a new multilocus sequence typing scheme

**DOI:** 10.1371/journal.pone.0200773

**Published:** 2018-07-30

**Authors:** Linda Grillová, Tanika Bawa, Lenka Mikalová, Angèle Gayet-Ageron, Kay Nieselt, Michal Strouhal, Patrice Sednaoui, Tristan Ferry, Matthias Cavassini, Stephan Lautenschlager, Fabrizio Dutly, Marta Pla-Díaz, Michael Krützen, Fernando González-Candelas, Homayoun C. Bagheri, David Šmajs, Natasha Arora, Philipp P. Bosshard

**Affiliations:** 1 Department of Biology, Masaryk University, Brno, Czech Republic; 2 Department of Fundamental Neuroscience, University of Geneva, Geneva, Switzerland; 3 Division of Clinical Epidemiology, University of Geneva Hospitals and Faculty of Medicine, Geneva, Switzerland; 4 Center for Bioinformatics, University of Tübingen, Tübingen, Germany; 5 Medical Biology, Institute Alfred Fournier, Paris, France; 6 Hospices civils de Lyon, Lyon, France; 7 Department of Infectious Diseases, Centre Hospitalier Universitaire Vaudois, Lausanne, Switzerland; 8 Department of Dermatology, Triemlispital, Zurich, Switzerland; 9 IMD Institut für medizinische & molekulare Diagnostik AG, Zurich, Switzerland; 10 Unidad Mixta Infección y Salud Pública FISABIO/Universidad de Valencia, CIBER in Epidemiology and Public Health, Valencia, Spain; 11 Department of Anthropology, University of Zurich, Zurich, Switzerland; 12 Repsol Technology Center, Madrid, Spain; 13 Institute for Evolutionary Biology and Environmental Studies, University of Zurich, Zurich, Switzerland; 14 Zurich Institute of Forensic Medicine, University of Zurich, Zurich, Switzerland; 15 Department of Dermatology, University Hospital Zurich, Zurich, Switzerland; 16 Faculty of Medicine, University of Zurich, Zurich, Switzerland; Defense Threat Reduction Agency, UNITED STATES

## Abstract

Syphilis is an important public health problem and an increasing incidence has been noted in recent years. Characterization of strain diversity through molecular data plays a critical role in the epidemiological understanding of this re-emergence. We here propose a new high-resolution multilocus sequence typing (MLST) scheme for *Treponema pallidum* subsp. *pallidum* (TPA). We analyzed 30 complete and draft TPA genomes obtained directly from clinical samples or from rabbit propagated strains to identify suitable typing loci and tested the new scheme on 120 clinical samples collected in Switzerland and France. Our analyses yielded three loci with high discriminatory power: TP0136, TP0548, and TP0705. Together with analysis of the 23S rRNA gene mutations for macrolide resistance, we propose these loci as MLST for TPA. Among clinical samples, 23 allelic profiles as well as a high percentage (80% samples) of macrolide resistance were revealed. The new MLST has higher discriminatory power compared to previous typing schemes, enabling distinction of TPA from other treponemal bacteria, distinction between the two main TPA clades (Nichols and SS14), and differentiation of strains within these clades.

## Introduction

*Treponema pallidum* subsp. *pallidum* (TPA), the bacterium responsible for syphilis, causes more than 5.6 million new syphilis cases annually and an increase in number of cases has been noted worldwide in the last few years [1]. The recent global outbreaks of syphilis infections are driven by multiple factors including suboptimal diagnostics in the early phase of infection, patients not adopting safer sex rules, mutations conferring macrolide resistance, and the lack of a syphilis vaccine [2]. However, a comprehensive epidemiological understanding of the underlying factors and the patterns characterizing the re-emergence is still missing. Molecular typing is required in order to discriminate genetic variants and investigate their potential association with phenotypic features such as pathogenicity and antibiotic resistance. Furthermore, molecular typing provides population structure data that can be used to map the geographical distribution of strain types, draw associations between strains and specific demographic groups, and examine transmission dynamics. In some cases, molecular typing can also be a valuable diagnostic tool, as illustrated by recent cases of syphilis-bejel confusion that were resolved through genetic data [3, 4].

The molecular characterization of TPA is limited by the lack of a continuous cultivation system under *in vitro* conditions. Therefore, research efforts have been primarily directed at strains propagated in laboratory animals, leading to the publication of a few TPA whole genomes since 1998 [5–10]. The data from these genomes as well as that of individual genes from clinical samples have been used in the development of molecular typing schemes (Table 1). Due to the low level of variation across TPA strains (below 0.03%), these schemes generally target the most variable loci, in order to gain sufficient discriminatory power. These loci include genes predicted to code for outer membrane proteins (TP0136, TP0548) or virulence factors (*tpr* genes), genes comprising tandem repeats (*arp* and *rps*A), as well as the genes associated with resistance to macrolides (23S rRNA genes) [15–17].

**Table 1:** Typing schemes for TPA bacteria (since 1998) and the new proposed MLST scheme.

Typing method	Genes/Loci	Methods	Year of introduction	References
CDC^a^ -typing (CDCT)	*arp*, *tpr*E, *tpr*G, *tpr*J	RFLP^b^ of *tpr* genes, determination of repeats in *arp* gene	1998	[11]
Sequencing-based molecular typing (SBMT)	TP0136, TP0548, 23S rRNA	Sanger sequencing	2006	[12]
Enhanced CDC^a^ -typing (ECDCT)	*arp*, *tpr*E, *tpr*G, *tpr*J, TP0548	RFLP^b^ of *tpr* genes, determination of repeats in *arp* gene, Sanger sequencing of TP0548	2010	[13]
CDC^a^ -typing with additional analyses of *rps*A gene (CDC-*rps*A)	*arp*, *rps*A, *tpr*E, *tpr*G, *tpr*J	RFLP^b^ of *tpr* genes, determination of repeats in *arp* and *rps*A genes	2010	[14]
Proposed MLST	TP0136, TP0548, TP0705, 23S rRNA	Sanger sequencing	2018	This study

^a^ Centers for Disease Control and Prevention, Atlanta, United States.

^b^RFLP—restriction fragment length polymorphism.

The most commonly used schemes are the CDCT and ECDCT with over 3,000 clinical isolates typed worldwide (reviewed in [18, 19]). However, these typing schemes were recently questioned due to possible intra-strain variability of the *arp* and *tpr* loci. These two loci were found to reveal subtype discrepancies in 11 out of 18 parallel samples taken from sero-positive individuals [20]. An alternative typing scheme, the SBMT, has been used to type over 170 clinical samples in Argentina, Belgium and the Czech Republic [12, 19, 21–23]. One of the limitations of all these typing schemes is that they generally have low discriminatory power for strains of the SS14-clade [19, 22], the more dominant of the two major clades identified among contemporary samples, and thus do not exploit the potential diversity present in current infections.

Recently, the availability of TPA genome-wide data from current infections have increased through the application of new enrichment methods to eliminate background DNA prior to the next generation sequencing (NGS) [24, 25]. While whole genome sequencing is not yet time and cost effective for the clinical setting, the availability of these new treponemal genomes provide us with the opportunity to more carefully examine diversity patterns and potential new typing loci in order to design the most up-to-date molecular typing method.

In this work, we aim to i) identify candidate typing loci based on the analyses of genome-wide data from TPA, ii) select loci providing the highest discriminatory power for inclusion in a new MLST typing scheme, and iii) apply the MLST to a set of 120 clinical samples from Switzerland and France collected between 2011 and 2015.

## Materials and methods

### Design of the typing scheme

We identified candidate loci for TPA typing in a dataset comprising 12 whole or draft genome sequences obtained from rabbit propagated reference TPA strains and 18 draft genomes sequenced directly from clinical specimens. These samples were collected in countries of three continents (Argentina, Austria, Czech Republic, Mexico, The Netherlands, USA, and Switzerland) in the period between 1912 and 2013 [6–10, 24] (S1 Table). The dataset contained sequences from both genetically distinct groups of TPA strains, SS14 clade (n = 21) and Nichols clade (n = 9), respectively. Since the *tpr* genes were shown to be among the most variable chromosomal loci [26] and the stability of *tpr*E, *tpr*G, and *tpr*J genes was recently questioned [20], we excluded all *tpr* genes from the analyses. Other paralogous regions, intergenic regions, genes with repetitions and sites with ambiguous data were excluded as well.

We selected candidate loci for TPA typing if; i) they contained at least four variable sites with a single nucleotide variant (SNV) density >0.001 (S2 Table), which is 10 times and 50 times higher than the average SNV density inside the Nichols-clade and SS14-clade, respectively, ii) they displayed at least two different haplotypes within each clade, and if iii) the SNVs were not too widespread in order to use as short as possible loci for increasing the amplification efficiency. We used MEGA6 [27] to visualize the sequence alignment and to identify parsimony-informative sites.

### Clinical samples, DNA isolation and qPCR

We performed the genetic analysis in 120 samples collected from 120 patients diagnosed with early syphilis or syphilis of unknown stage between September 2011 and May 2015 in outpatient clinics in five cities in Switzerland and France (Zurich, n = 70; Geneva, n = 10; Lausanne, n = 2; Lyon, n = 21; and Paris, n = 17). The samples included 119 genital, anal, oral or rectal lesion swabs and 1 tissue sample. DNA isolation and routine real-time PCR for diagnosis have been performed as described previously [28, 29] (detailed in the Supplementary Methods). We had clinical data about the patient age, gender, site of infection, HIV status and syphilis stage for 93/120 patients.

### Nested PCR and DNA sequencing

Five candidate loci (TP0136, TP0462, TP0548, TP0705 and TP0865) and the 23S rRNA genes were amplified by a nested-PCR approach with a touchdown protocol in the first PCR reactions (primer sequences and conditions detailed in S1 Supplementary Methods and S3 Table). PCR products were purified using a QIAquick PCR Purification Kit (Qiagen, Hilden, Germany) according to the manufacturer’s instructions and sequenced on an automated capillary DNA sequencing system (GATC-Biotech AG, Constance, Germany). Sequence analyses were performed using the Lasergene software (DNASTAR v. 7.1.0.; DNASTAR, Madison, WI). Sequences representing allelic variants in different typing loci were deposited in GenBank under the following accession numbers: MG894082—MG894119 (S4 Table). For the 23S rRNA genes, positions corresponding to 2058 and 2059 in the *Escherichia coli* 23S rRNA gene (accession no. V00331) were checked for A→G mutations indicative of macrolide resistance [15–17, 30–31].

### Comparison of typing schemes

We analyzed the number of 60 bp-long repetitions in the *arp* gene and amplified the *tpr*E, G, and J genes as described previously [11, 13] with minor modifications for the *tpr* genes, including an increase of the extension temperature from 68°C to 72°C and the use of GoTaq Flexi DNA polymerase (Promega, Mannheim, Germany). The information revealed from the RFLP of the *tpr* genes, determination of repeats in the *arp* gene and sequencing of TP0136, TP0548 and 23S rDNA loci allowed us to compare the typing efficiency of the currently used typing schemes with the MLST scheme proposed in this study.

### Typing stability

In order to test the typing stability of the new typing scheme, we amplified all loci in eleven passages of the TPA reference strain DAL-1, which was continuously propagated in rabbits during 142 days (representing 114 TPA generations). Moreover, we tested the concordance in five samples collected in the Czech Republic during 2004–2010 and pertaining to five epidemiologically related patients.

### Phylogenetic analyses

We generated maximum likelihood (ML) phylogenetic trees with MEGA 6 [27] using the Tamura Nei model and 1000 pseudorandom bootstrap replicates. Median-joining (MJ) networks were generated with Network version 4 [32]. We created genome-wide data MJ networks for the 30 samples published in [24] using 768 variable positions (including putative recombinant loci and excluding positions with missing data). The MJ networks for the candidate typing loci included 87 variable positions (5 candidate loci) and 74 variable positions (3 candidate loci). Deletions and insertions were counted as single events. The software Sequence Matrix 1.8 was used for the sequence concatenations [33].

### Statistical methods

We had a fixed number of samples (n = 120) allowing us to detect medium effect size associations between some genetic and clinical characteristics. Genetic characteristics of clinical isolates (macrolide resistance, full allelic profiles, allelic variants per gene and clade) were examined to test for associations with clinical characteristics such as patient’s origin, MSM status, age, gender, HIV infection, serological results and syphilis stage (as detailed in the S1 Supplementary Methods). Statistical analyses were carried out in RStudio (version 1.1.383) using Fischer’s exact tests for categorical variables and Kruskal Wallis test for continuous variables. Mean nucleotide diversity (π) was calculated with MEGA 6 [27].

### Ethics statement

This study was approved by the ethics committees of Zurich (2016–01518), Lausanne (380/11), Geneva (11–151) and Lyon (L11-150). All patients provided informed written consent.

## Results

### Candidate typing loci and their discriminatory power

Based on the selection criteria, we identified five genes as candidates for TPA typing: TP0136, TP0462, TP0548, TP0705 and TP0865. TP0136 had the largest SNV density across all genes examined, followed by TP0462, TP0548, TP0865 and TP0705 (S2 Table). Four of these loci code for outer membrane proteins and one for penicillin-binding protein. All but one (TP0705) have been previously identified as putative recombinant genes [24, 34, 35]. To assess the resolution power of the five candidate loci, we compared the MJ network built from their concatenated sequences (incorporating 87 variable positions) to the genome-wide data network (incorporating 768 variable positions) for 30 draft and complete genome sequences (S1 Table). The concatenated sequences of the five loci were able to distinguish 8/26 (30.8%) haplotypes identified using genome-wide data (data not shown). By only using three of the five candidate loci (TP0136, TP0548 and TP0705), the same resolution power as with all five loci was achieved.

**Fig 1:**
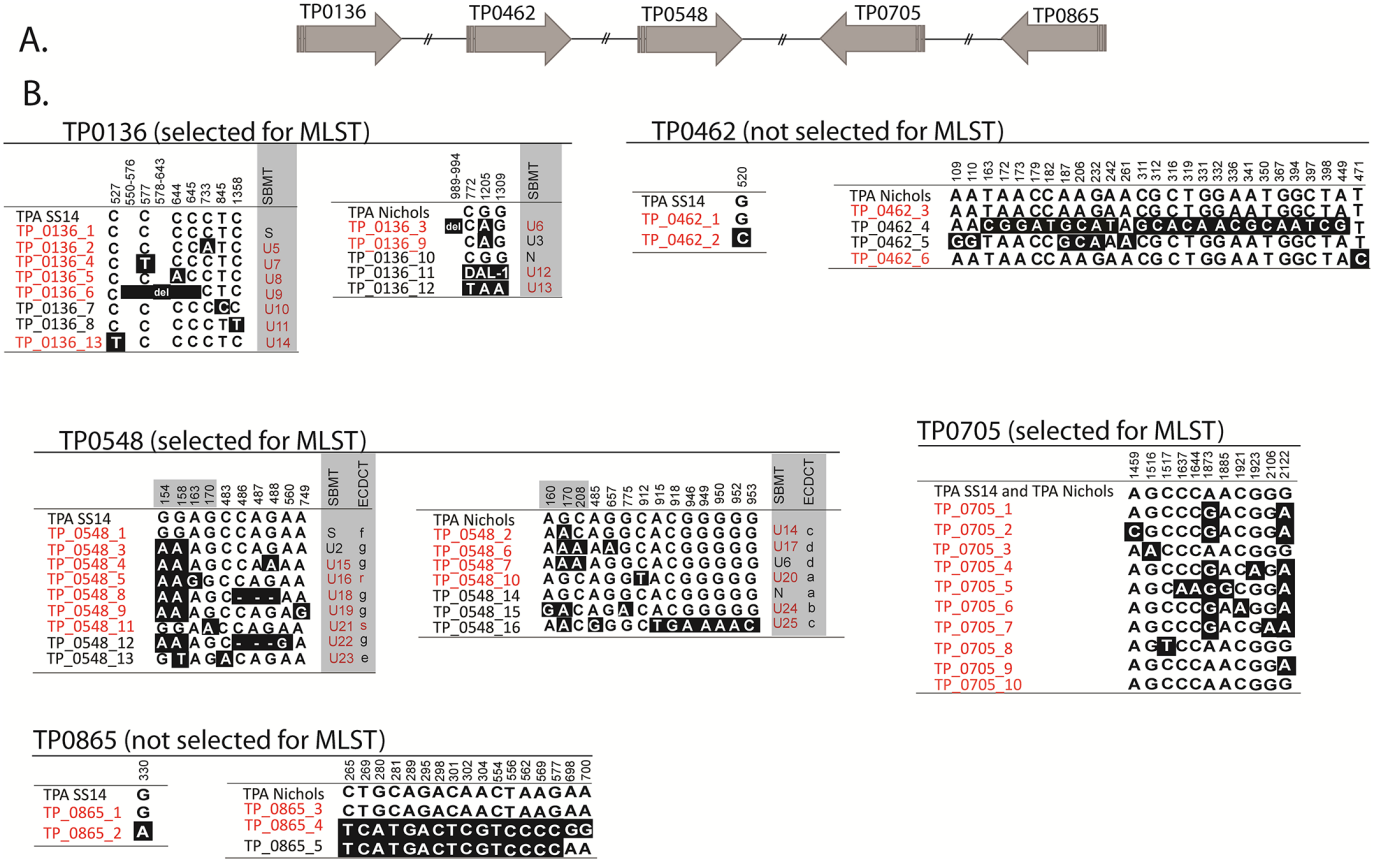
Allelic variants of all available strain sequences for TP0136, TP0462, TP0548, TP0705 and TP0865. Alleles found in 120 clinical samples examined in this study (samples from France and Switzerland) and in 63 samples published in [24, 25, 36] (total n = 183) are shown. **A. Structural arrangement of candidate loci on the genome.** The TP0705 and TP0865 genes lie on the complementary DNA chain. **B. Allelic variants**. Sequence alignments show only positions containing nucleotide variants. Numbers correspond to nucleotide positions in TPASS_0136, TPASS_0462, TPASS_0548, TPASS_0705, TPASS_0865 genes (TPA SS14; CP004011.1) or to TPANIC_0136, TPANIC_0462, TPANIC_0548, TPANIC_0705, TPANIC_0865 genes (TPA Nichols; CP004010.2). Deletions are shown with dashes or “del”. Allele variants that were found among clinical samples in this study (clinical samples from France and Switzerland) are shown in red. For TP0136 and TP0548, the sequence variants were translated to both current molecular typing systems (SBMT and ECDCT) and new sequence variants, which were not identified in previous typing studies, are shown in red. Sequence variant TP0136_11 represent TPA strain Dallas (DAL-1), which contains 30 SNVs and a 58 bp long deletion in TP0136 locus.

**Fig 2:**
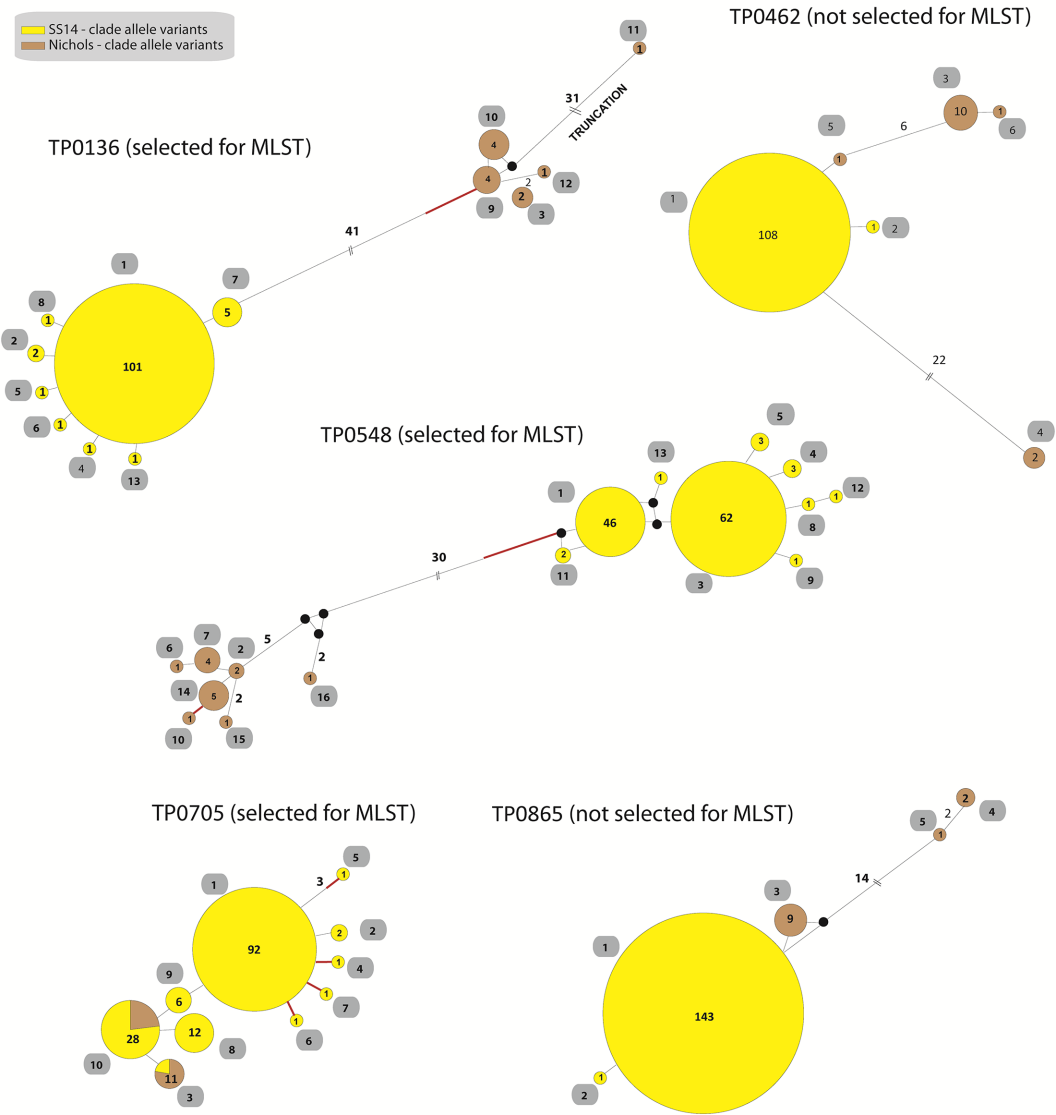
Median joining networks of all available strain sequences for TP0136, TP0462, TP0548, TP0705 and TP0865. Alleles found in 120 clinical samples examined in this study (samples from France and Switzerland) and in 63 samples published in [24, 25, 36] (total n = 183) are shown. Median joining networks per locus show the different allelic variants and the number of mutational differences among them. Number of mutations, when above one, is given close to branches. Red branch indicates the mutation that did not result in amino acid replacements. Inferred allelic variants (median vectors) are shown as black connecting dots. If contiguous, indels were considered as a single event only. Yellow circles represent the SS14-clade and brown circles represents the Nichols-clade. The number found among the 183 samples is shown inside the circles. Allelic variant numbering as proposed for the MLST scheme are shown in the grey fields.

We further analyzed the five candidate loci in a combined dataset of all (n = 63) previously published sequences [24, 25, 36] and of the sequences of the 120 qPCR-positive clinical samples from this study. In this combined sequence dataset, 16, 13, 10, 6 and 5 different allele variants were found in the TP0548, TP0136, TP0705, TP0462 and TP0865 loci, respectively (Figs 1 and 2). For the SS14-clade, the highest variability was found in TP0705, followed by TP0548, TP0136, TP0462 and TP0865 loci, while for the Nichols-clade, the highest variability was found in TP0548 followed by TP0136, TP0462, TP0865 and TP0705 loci. A much higher nucleotide mean diversity was found within the Nichols-clade (π = 0.0121) when compared to SS14-clade strains (π = 0.0002).

The TP0136 and TP0548 loci clearly distinguished between the SS14 and Nichols genetic groups with 41 and 30 parsimony-informative sites, respectively. In contrast, the TP0462 and TP0865 loci revealed different haplotypes for the two genetic groups but no parsimony-informative sites distinguishing them. The TP0705 locus shared identical alleles among strains from SS14- and Nichols-clades.

### A new multilocus sequence typing (MLST) scheme for molecular typing

To keep the number of typing loci minimal, we propose MLST of the three loci TP0136, TP0548 and TP0705 as a new typing scheme of TPA. The MLST scheme is based on different allelic profiles (haplotypes) described by a three-letter code, where the first number corresponds to the TP0136 allele, the second to the TP0548 allele and the third to the TP0705 allele (e.g. 1.3.1).

### MLST and prevalence of macrolide resistance in clinical samples from France and Switzerland

We analyzed 120 clinical samples with the new MLST. Clinical characteristics of the patients are depicted in Table 2. Ninety-seven (80.8%) samples were PCR-positive for at least one of the three typing loci, and 54 (45%) of them could be fully typed. We identified eight allele variants for TP0136, 11 for TP0548 and ten for TP0705 (Fig 1B, shown in red). In total, we found 23 different allelic profiles (18 within the SS14-clade, four within the Nichols-clade and one undetermined) (Table 3). The allelic profiles 1.1.1 and 1.3.1 (pertaining to the SS14-clade) were the most frequent profiles and together accounted for 67% of fully typed samples with 1.3.1 being the only profile detected every year (2011–2015) in all five cities involved in this study. Only 9/97 samples (9.3%) belonged to the Nichols-clade.

**Table 2:** Clinical characteristics of patients with syphilis.

Variable	Patients (n = 93)^a^
Mean age, yr	41.7
Sex, n (%)	M 92 (98.9); F 1 (0.1)
MSM, n (%)^b^	63/74 (85.1)
HIV infection, n (%)^c^	30/76 (39.5)
Materials ^d^	
Genital lesion	26 (31.7)
Rectal lesion	47 (57.3)
Anal lesion	1 (1.2)
Oral lesion	7 (8.5)
Tissue	1 (1.2)
Stage of syphilis, n (%)	
Primary	32 (34.4)
Secondary	10 (10.8)
Syphilis without further characterization	51 (54.8)

^a^Information on clinical characteristics was unavailable for 27 clinical samples.

^b^There is no information about sexual orientation for 18 samples.

^c^There is no information about HIV status for 17 samples.

^d^There is no information about material for 11 samples.

**Table 3:** MLST allelic profiles for fully and partially typed Swiss and French clinical samples (n = 97; for partially typed samples, only new allelic profiles are shown).

Allelic profile^a^	Typing	TP_0136 allelic variant^b^	TP_0548 allelic variant^b^	TP_0705 allelic variant^b^	23S rRNA^c^	Genetic group	No. of samples
1.1.1	Complete	1	1	1	S (60%)/R8 (40%)	SS14-clade	11
1.1.3	Complete	1	1	3	R9	SS14-clade	2
1.11.8	Complete	1	11	8	NA^d^	SS14-clade	1
1.3.1	Complete	1	3	1	S (5%) /R8 (95%)	SS14-clade	25
1.3.7	Complete	1	3	7	R8	SS14-clade	1
1.4.1	Complete	1	4	1	R8	SS14-clade	3
1.5.1	Complete	1	5	1	R8	SS14-clade	2
1.8.1	Complete	1	8	1	R8	SS14-clade	1
1.9.1	Complete	1	9	1	R8	SS14-clade	1
13.1.1	Complete	13	1	1	S	SS14-clade	1
2.1.2	Complete	2	1	2	R8	SS14-clade	1
3.2.3	Complete	3	2	3	R8	Nichols-clade	2
4.3.1	Complete	4	3	1	NA^d^	SS14-clade	1
5.3.8	Complete	5	3	8	NA^d^	SS14-clade	1
6.3.1	Complete	6	3	1	S	SS14-clade	1
1.X.9	Partial	1	NA^d^	9	R8	SS14-clade	-^e^
X.10.X	Partial	NA^d^	10	NA^d^	R8	Nichols-clade	-^e^
X.3.5	Partial	NA^d^	3	5	R8	SS14-clade	-^e^
X.6.X	Partial	NA^d^	6	NA^d^	NA^d^	Nichols-clade	-^e^
X.7.3	Partial	NA^d^	7	3	S	Nichols-clade	-^e^
X.X.10	Partial	NA^d^	NA^d^	10	S	NA^3^	-^e^
X.X.4	Partial	NA^d^	NA^d^	4	R8	SS14-clade	-^e^
X.X.6	Partial	NA^d^	NA^d^	6	R8	SS14-clade	-^e^

^a^Allelic profiles are based on a three-number code: the first number corresponds to the allelic variant in TP0136 locus, the second corresponds to the allelic variant in the TP0548 locus and the third corresponds to the allelic variant in the TP0705 locus. We used X to denote that the allelic variant was not determined.

^b^The SNVs that determine the allelic variant are shown in the Fig 1.

^c^ S—no mutation in 23S rDNA (sensitive), R8 –A2058G mutation in 23S rDNA (resistance), R9 –A2059G mutation in 23S rDNA (resistance). When sensitive and resistant cases were found for one profile, the frequency is given in parenthesis.

^d^ NA, not available.

^e^ For partially typed samples the no. of samples is not shown.

Apart from the candidate loci, we analyzed the 23S rRNA gene operons to check for macrolide susceptibility/resistance. The operons were successfully amplified in 79 samples. In 63 (79.7%) of them, we either found the A2058G (60 samples, 95.2%) or the A2059G (3 samples, 4.8%) mutation indicating resistance. No strain harbored both mutations. Macrolide resistance was present in both the SS14-clade and Nichols-clade (81% and 60%, respectively) and was relatively high in all tested locations including Lausanne (100%), Paris (85.7%), Lyon (83.3%), Zurich (80%) and Geneva (62.5%). The allelic variant 1.3.1, which is part of the SS14-clade, was associated with macrolide resistance (*P* = 0.0006). We further search for associations between allelic profiles and clinical characteristics of patients. Besides the association between clade and city (all Nichols-clade strains were found in Zurich), no others were found.

### Comparison of MLST with other typing schemes

While MLST revealed 23 different allelic profiles among 97 typeable clinical samples (Table 3), SBMT revealed 19 different genotypes among 99 typeable samples, of which 12 have not been observed in previous studies (S5 Table). With the ECDCT scheme, 89 samples could be typed revealing 15 different subtypes (S6 Table). When comparing only fully typed samples, we identified 15 allelic profiles, 11 genotypes and eight subtypes with MLST, SBMT and ECDCT, respectively. The ECDCT subtype 14d/g was further divided into five allelic profiles with MLST. Based on *arp* repeat sizes and *tpr* restriction fragment length polymorphisms, the MLST allelic profiles 1.1.1 and 1.3.1 were divided into two and four different ECDCT subtypes, respectively.

**Fig 3:**
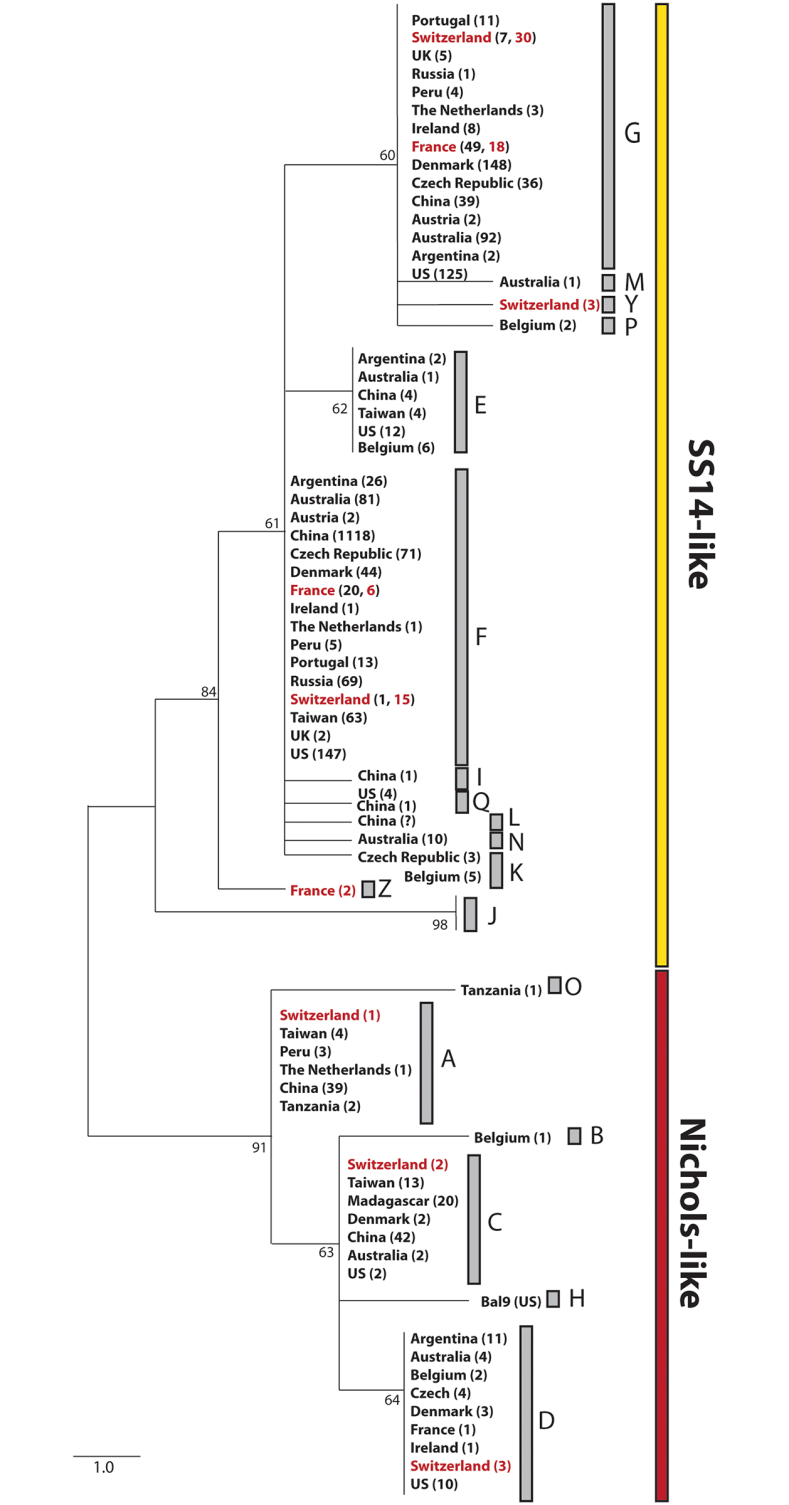
Phylogeny for sequences obtained in typing studies. Maximum likelihood tree produced in MEGA 6 for the 83 bp overlapping fragment of the TP0548 gene typed with MLST, ECDCT and SBMT. The letters A-S stand for the classification in ECDCT and SBMT typing studies. Rabbit passed strains BAL9 was included as representative for type “H”, which was not found among clinical samples yet. For subtype”L” the number of identified samples is not known [37]. Subtype”J” does not correspond to TPA but was found in *T*. *pallidum* subsp. *endemicum* and *T*. *pallidum* subsp. *pertenue* [35, 38]. As observed, TP0548 does not distinguish TPA from TEN or TPE, reflecting potential recombination events between these three. All samples were obtained directly from patients except the BAL9 (passed through rabbits) and isolates from Tanzania (isolated from flies). The numbers in the brackets represent the number of samples identified in the given country. The numbers in red represent the Swiss and French samples from this study.

The three typing schemes MLST, ECDCT and SBMT share an 83 bp overlapping fragment of the TP0548, enabling comparative analyses of samples typed across the globe with these schemes. As illustrated in Fig 3, a phylogenetic tree revealed that most of the Swiss and French samples grouped together with seven out of 19 previously identified subtypes. Interestingly, two Swiss samples and two French samples produced two new subtypes within the SS14 clade, respectively, not observed in other studies. These two new subtypes are defined as MLST TP0548 alleles 5 and 11 (and designated as Y and Z in the ECDCT nomenclature).

### Typing stability and epidemiological concordance

In order to determine the typing stability of MLST, we analyzed eleven passages of the TPA reference strain DAL-1 that was continuously propagated in rabbits during 142 days (representing 114 TPA generations). All passages revealed the same allelic profile. In addition, samples of five epidemiologically related patients were typed with MLST; we did not find any discrepancies in allelic profiles.

## Discussion

Syphilis is an important public health problem and an increasing incidence has been noted in recent years. Efficient and affordable typing methods that can be performed in any laboratory around the world with access to sequencing facilities are warranted. These methods would provide information on the frequency of strain types and changes throughout time, on whether particular strain types are associated with specific patient groups, and on patterns among antibiotic resistant strains. With the introduction of TPA enrichment and WGS directly from clinical samples in 2016 [24, 25] the number of available TPA whole genomes increased, opening an opportunity to identify new genes suitable for typing.

We analyzed 30 whole or draft genome TPA sequences to propose a new, efficient and easy-to-perform molecular typing scheme. Taking into account that TPA is an uncultured pathogen and that clinical samples contain low quantities of pathogen DNA, our aim was to keep the number of typing loci minimal. At the same time, considering that TPA is classified as a monomorphic bacterium and each SNV may be informative, we targeted loci with the highest SNV density to ensure sufficient variability to capture overall population diversity.

Our results reveal three variable genes suitable for typing (TP0136, TP0548 and TP0705) which, together with the 23S rRNA genes, are here proposed as a new molecular typing method for syphilis. The proposed TPA MLST shares two typing loci (TP0136, and TP0548) with a recently published MLST for a close relative of TPA—*Treponema pallidum* subsp. *pertenue* [38], as well as with the previously published TPA SBMT [12, 21]. Thus, MLST can be considered as enhanced SBMT with additional analysis of TP0705. This latter gene codes for a penicillin-binding protein. TP0136 and TP0548 code for outer membrane proteins, with TP0136 products binding to host fibronectin in the extracellular matrix and playing an important role in local replication [34]. While both TP0136 and TP0548 have been identified as putative recombinant loci, the clustering patterns within TPA recapitulate the distinction between the SS14 and Nichols clade observed with genome-wide data, and are thus congruent with vertical relationships among TPA strains. This congruence may result from recombination not occurring within TPA, but between TPA and *Treponema pallidum* subsp. *endemicum* (TEN) or *Treponema pallidum* subsp. *pertenue* (TPE) (for TP0548 see Fig 3) [24, 35]. Nonetheless, the proposed MLST is based on allelic profiles in order to account for potential horizontal gene exchange. As with other MLST schemes, the allelic profiles can easily be converted to the corresponding nucleotide sequence data.

The new MLST reveals high resolution power (more than 30% of the resolution achieved with genome-wide data), allowing the distinction of TPA from other treponemal subspecies (S1 Fig), the distinction between the two TPA clades SS14 and Nichols (TP0136, TP0548), and the differentiation of strains within each of these clades (TP0548 for the Nichols clade, and TP0705 for the SS14 clade). When designing a new MLST it is important to find a balance between increasing discriminatory power and limiting the number of samples from which one can get complete typing data. The amplification efficiency of typing schemes is dependent on several factors, e.g. type of material, treatment history of patients, time between sampling and DNA isolation, DNA extraction method, length of the amplification product, and amplification protocol. In order to optimize the resolution power, we opted for relatively long loci that resulted in an overall moderate rate of 45% of fully typed samples. This number is comparable to the amplification efficiency of recently published TP typing studies (ranging from 15.3% to 97% with a median of 46.9% [19, 23, 39–43]. During the course of our study, we further optimized the PCR protocol (see Supplementary Methods) to increase the amplification efficiency (data not shown). Unfortunately, we did not have enough material left in many of the samples, thus, we did not have the opportunity to apply this new enhanced protocol to the whole set of samples. However, when the enhanced protocol was applied to a subset of 30 samples, the amplification efficiency reached 77%. For this reason, we are expecting higher amplification efficiency in the future. The CDCT system has revealed different *arp* and *tpr* subtypes in parallel samples of whole blood and genital smears taken from the same patient [20] indicating inconsistent typing stability. Furthermore, CDCT subtypes, which are based on sequence length differences, do not provide phylogenetic information that could be used to track syphilis transmission dynamics.

The application of the different typing schemes to the Swiss and French dataset showed that MLST yields the highest discriminatory power, with 23 different allelic profiles among 97 typeable samples. Eighteen belonged to the SS14-clade, four to the Nichols-clade and one could not be assigned because in this case only TP0705 data were available. Only nine samples (9.3%) were part of the Nichols-clade, all of which were detected in Zurich. The relative frequency of Nichols and SS14 strains is consistent with that found in other studies [reviewed in 19, 24]. However, a much higher prevalence of samples belonging to the Nichols-clade was reported in Peru (21.4%) [39], Taiwan (20.2%) [40], Madagascar (100%) [13] and Argentina (26.8%) [19].

We found an alarmingly high percentage of macrolide resistance strains (80% of samples), consistent with other studies performed in Australia [41], Cuba [42], China [43], USA [14] and Europe including the Czech Republic [22], Ireland [44], England [45], Belgium [23], Portugal [25], the Netherlands and Austria [24]. Accordingly, azithromycin should no longer be recommended as second line therapy for patients with penicillin allergy or bleeding disorders [46]. Of the two dominant allelic profiles found in this study, 1.3.1 was associated with macrolide resistance. Only a limited number of associations between genetic variants and patient characteristics have been identified so far. A study by Marra and colleagues published in 2010 [13] found that 50% of patients in Seattle with ECDC subtype 14d/f had neurosyphilis. Other studies have found correlations between genotypes and patient’s age, origin, MSM-status and serofast status [19, 22, 47]. However, we did not observe any associations for the Swiss and French samples other than the location of Nichols clade samples, which were all collected in Zurich.

In this study, we present MLST as a new molecular typing scheme for syphilis based on the sequencing of three loci (TP0136, TP0548 and TP0705), and the additional analysis of the 23S rDNA genes to determine macrolide resistance/sensitivity. As demonstrated in this study, the novel MLST scheme yields higher discriminatory power compared to previous typing schemes. We encourage the scientific community and public health authorities to use this MLST method, which can be easily and quickly performed in standard laboratories We expect this tool to open new opportunities in epidemiology allowing longitudinal studies of TPA allelic profiles in different locations, better tracking of syphilis infections, and finding associations of particular strains with specific patient groups, all in favor of a better understanding of the epidemiology of syphilis.

## Supporting information

S1 Supplementary Methods (DOCX)

S1 Table Draft and complete genome sequences (n = 30) used for identification of candidate loci. (DOCX)

S2 Table Genes with the highest SNV density among samples listed in S1 Table (n = 30). Intergenic regions, paralogous genes (including *tpr* genes), genes with repetitions and sites with ambiguous data were not included in this analysis. Only genes containing at least four variable sites resulting in the SNV density > 0.001 are shown. Candidate loci are shown in bold.
(DOCX)

S3 Table Primers used for the nested-PCR amplification for the candidate loci and the 23S rRNA gene. (DOCX)

S4 Table GenBank accession numbers corresponding to the particular allelic variants. (DOCX)

S5 Table Nineteen different genotypes found among 99 typeable Swiss and French clinical samples by Sequencing-based molecular typing (SBMT). (DOCX)

S6 Table Fifteen different subtypes found among 89 typeable Swiss and French clinical samples by ECDCT. (DOCX)

S1 Fig Phylogeny of MLST allelic profiles obtained from complete genomes representing different *Treponema pallidum* subspecies. Maximum likelihood tree produced in MEGA 6 for concatenated sequences of typing loci (TP_0136, TP_0548, TP_0705) in available complete genomes of reference strains representing different *Treponema pallidum* subspecies (TPA–*Treponema pallidum* subsp. *pallidum*; TPE—*Treponema pallidum* subsp. *pertenue*; TEN—*Treponema pallidum* subsp. *endemicum*).
(TIF)
