# Supplementary material for: Molecular characterization of Treponema pallidum subsp. pallidum in Switzerland and France with a new multilocus sequence typing scheme
Source: PLoS One. 2018 Jul 30;13(7):e0200773. doi: 10.1371/journal.pone.0200773 (PMC6066202; doi:10.1371/journal.pone.0200773)
Supplement: S1 Supplementary Methods — (DOCX) [file pone.0200773.s001.docx]

**Supplementary Methods**

**Clinical samples.** Clinical samples (n=120) were collected from patients diagnosed with syphilis from four different hospitals in Switzerland (Hospitals and Faculty of Medicine, University of Geneva, Geneva; Department of Infectious Diseases, Centre Hospitalier Universitaire Vaudois, Lausanne; Department of Dermatology, Triemlispital, Zurich; Department of Dermatology, University Hospital Zurich, Zurich), a private laboratory in Switzerland (IMD Institut für medizinische & molekulare Diagnostik AG, Zurich), one hospital in Lyon, France (Hospices civils de Lyon, Lyon) and one sexually-transmitted diseases outpatient clinic in Paris, France (Medical Biology, Institute Alfred Fournier, Paris).

**DNA isolation of samples collected in Zurich**. A total of 0.5 ml of swab supernatants were centrifuged at 14 000 rpm for 10 min. The resulting pellets were incubated with 40 μg proteinase K (Roche Diagnostics, Rotkreuz, Switzerland) at 54°C with 300 rpm for 2 h. After proteinase K inactivation (94°C for 10 min) the samples were centrifuged at 14 000 rpm for 10 min and the supernatants were used for further analyses [1].

**DNA isolation of samples collected in Paris, Lausanne, Lyon and Geneva.** DNA was isolated from 400 µl of swab extracts. Mechanical lysis (glass beads <10^6^ µm; Sigma, Buchs, Switzerland) was performed with a mickle apparatus during 5 minutes. The supernatant was then incubated 30 minutes at 37°C with a lytic enzyme cocktail (4µl of LYT030 = N-acetylmuramidase + zymolase in a glycerol/Tris-Acetate buffer) and a detergent bacterial lysis buffer (160µl of rMBF buffer; Roche Diagnostics GmbH, Mannheim, Germany). The samples were processed with the MagNa Pure compact system (Roche Diagnostics GmbH, Mannheim, Germany) using the nucleic acid isolation kit I according to the "blood" protocol, yielding an elution volume of 100µl [2].

**qPCR.** Real-time PCR targeting the 16S rRNA gene or 47 kDa membrane lipoprotein gene was used for TPA DNA quantification as described previously [1, 2].

**Nested PCR.** Six TPA loci (TP0136, TP0462, TP0548, TP0705, TP0865 and 23S rDNA) were amplified in the 120 clinical samples by nested-PCR. The 25 μl reaction mixtures of the first step of nested PCR contained 200 μM of dNTP (delivered with PrimeSTAR GLX DNA polymerase; Takara Bio Europe, Saint Germain en Laye, France), 5 μl of 5x GLX buffer, 9.5 μM of each primer, 0.625 U of PrimeSTAR GLX DNA polymerase (Takara Bio Europe, Saint Germain en Laye, France), and 1 μl of DNA. A touchdown protocol was used for the first PCR at the following conditions: initial denaturation at 94°C for 1 min; 8 cycles: 98°C for 10 s, 68°C for 15 s (annealing temperature gradually reduced by 1°C/every cycle), and 68°C for 1 min and 45 s; 35 cycles: 98°C for 10 s, 61°C for 15 s, and 68°C for 1 min and 45s (43 cycles in total); followed by the final extension at 68°C for 7 min. The second PCR was performed as described previously [3]. Briefly, each PCR mixture contained 200 μM deoxynucleotide triphosphate (dNTP) mixture, 2.5 μl of ThermoPol Reaction buffer, 25 μM of each primer, 0.25 U of Taq polymerase (New England BioLabs, Ipswich, MA, USA), and 1 μl of PCR product obtained from the first step of nested PCR. The reaction mixture was supplemented with PCR-grade water to a final volume of 25 μl. PCR amplification of all loci in the second step was performed under the following cycling conditions: 94°C (1 min); 94°C (30 s), 50°C (30 s), and 72°C (1 min, 45 s) for 40 cycles; and 72°C (7 min). Primer sequences are shown in the Supplementary Table 3.

**Statistical methods.** Genetic characteristics of clinical isolates were examined to test for associations with clinical characteristics of patients. RStudio (version 1.1.383) was used to run Fisher’s exact tests for categorical variables and Kruskal Wallis test for continuous variables. Genetic characteristics comprised sensitivity/resistance to macrolide antibiotics, full allelic profiles (e.g. 1.3.1), allelic variants per gene (e.g. TP0136_1, TP0548_3, TP0705_1) and the genetic clade (SS14 or Nichols-clade). The clinical characteristics comprised patient’s origin (Geneva, Lausanne, Lyon, Paris, Zurich), sexual orientation (MSM, heterosexual), age (as categorical or as a continuous variable), gender (male or female), HIV infection (positive or negative), serological results (positive or negative) and syphilis stage (primary or secondary). When using age as a categorical variable, we divided the patients into seven different age categories: 20-25 (1), 26-30 (2), 31-35(3), 36-40 (4), 41-50 (5), 51-60 (6), above 60 (7).

**Supplementary References**

1. Glatz M, Juricevic N, Altwegg M, *et al.* A multicenter prospective trial to asses a new real-time polymerase chain reaction for detection of *Treponema pallidum*, herpes simplex-1/2 and *Haemophilus ducreyi* in genital, anal and oropharyngeal ulcers. Clin Microbiol Infect. 2014; 20: 1020-1027.
2. Gayet-Ageron A, Ninet B, Toutous-Trellu L, et al. Assessment of the real-time PCR test to diagnose syphilis from diverse biological samples. Sex Transm Infect. 2009; 85(4): 264-269.
3. Matějková P, Flasarová M, Zákoucká H, et al. Macrolide treatment failure in a case of secondary syphilis: a novel A2059G mutation in the 23S rRNA gene of *Treponema pallidum* subsp. *pallidum*. J Med Microbiol. 2009; 58(6): 832–836.
