# Supplementary material for: Molecular characterization of Treponema pallidum subsp. pallidum in Switzerland and France with a new multilocus sequence typing scheme
Source: PLoS One. 2018 Jul 30;13(7):e0200773. doi: 10.1371/journal.pone.0200773 (PMC6066202; doi:10.1371/journal.pone.0200773)
Supplement: S1 Table — (DOCX) [file pone.0200773.s002.docx]

**Table S1. Draft and complete genome sequences (n=30) used for identification of candidate loci.**

| Sample Name | Genetic group | Location | Year of isolation | Source | Genome (IDs) | References |
| --- | --- | --- | --- | --- | --- | --- |
| Nichols | Nichols-clade | USA | 1912 | rabbit inoculation | Complete (CP004010.2) | [9] |
| SS14 | SS14-clade | USA | 1977 | rabbit inoculation | Complete (CP004011.1) | [9] |
| Chicago | Nichols-clade | USA | 1951 | rabbit inoculation | Complete (CP001752.1) | [6] |
| Mexico A | SS14-clade | Mexico | 1953 | rabbit inoculation | Complete (CP003064.1) | [7] |
| DAL-1 | Nichols-clade | USA | 1991 | rabbit inoculation | Complete (CP003115.1) | [8] |
| SEA81-4 | Nichols-clade | USA | 1981 | rabbit inoculation | Complete (CP003679.1) | [10] |
| AR2 | SS14-clade | Argentina | 2013 | clinical acquired | Draft^1^ | [24] |
| AU13 | SS14-clade | Austria | 2013 | clinical acquired | Draft^1^ | [24] |
| AU15 | SS14-clade | Austria | 2013 | clinical acquired | Draft^1^ | [24] |
| AU16 | SS14-clade | Austria | 2013 | clinical acquired | Draft^1^ | [24] |
| AU17 | SS14-clade | Austria | 2013 | clinical acquired | Draft^1^ | [24] |
| CZ27 | SS14-clade | Czech Republic | 2012 | clinical acquired | Draft^1^ | [24] |
| CZ33 | SS14-clade | Czech Republic | 2013 | clinical acquired | Draft^1^ | [24] |
| GRA2 (Grady) | SS14-clade | USA | 1980-1999 | rabbit inoculation | Draft^1^ | [24] |
| NE12 | SS14-clade | The Netherlands | 2013 | clinical acquired | Draft^1^ | [24] |
| NE13 | SS14-clade | The Netherlands | 2013 | clinical acquired | Draft^1^ | [24] |
| NE14 | SS14-clade | The Netherlands | 2013 | clinical acquired | Draft^1^ | [24] |
| NE15 | SS14-clade | The Netherlands | 2013 | clinical acquired | Draft^1^ | [24] |
| NE17 | SS14-clade | The Netherlands | 2013 | clinical acquired | Draft^1^ | [24] |
| NE19 | SS14-clade | The Netherlands | 2013 | clinical acquired | Draft^1^ | [24] |
| SW1 | SS14-clade | Switzerland | 2012 | clinical acquired | Draft^1^ | [24] |
| SW4 | SS14-clade | Switzerland | 2012 | clinical acquired | Draft^1^ | [24] |
| SW6 | SS14-clade | Switzerland | 2012 | clinical acquired | Draft^1^ | [24] |
| SW8 | SS14-clade | Switzerland | 2012 | clinical acquired | Draft^1^ | [24] |
| UW249B | SS14-clade | USA | 2004 | rabbit inoculation | Draft^1^ | [24] |
| BAL3 | Nichols-clade | USA | 1973 | rabbit inoculation | Draft^1^ | [24] |
| BAL73 | Nichols-clade | USA | 1973 | rabbit inoculation | Draft^1^ | [24] |
| NE20 | Nichols-clade | Netherlands | 2013 | clinical acquired | Draft^1^ | [24] |
| NIC2 (Nichols Seattle) | Nichols-clade | USA | 1912 | rabbit inoculation | Draft^1^ | [24] |
| SEA86-1 | Nichols-clade | USA | 1986 | rabbit inoculation | Draft^1^ | [24] |

^1^A minimum of 80% genome width covered by at least three reads with the Illumina sequencing platform.
