# Supplementary material for: Molecular characterization of Treponema pallidum subsp. pallidum in Switzerland and France with a new multilocus sequence typing scheme
Source: PLoS One. 2018 Jul 30;13(7):e0200773. doi: 10.1371/journal.pone.0200773 (PMC6066202; doi:10.1371/journal.pone.0200773)
Supplement: S2 Table — Intergenic regions, paralogous genes (including tpr genes), genes with repetitions and sites with ambiguous data were not included in this analysis. Only genes containing at least four variable sites resulting in the SNV density > 0.001 are shown. Candidate loci are shown in bold. (DOCX) [file pone.0200773.s003.docx]

**Table S2. Genes with the highest SNV density among samples listed in S1 Table (n=30).** Intergenic regions, paralogous genes (including *tpr* genes), genes with repetitions and sites with ambiguous data were not included in this analysis. Only genes containing at least four variable sites resulting in the SNV density > 0.001 are shown. Candidate loci are shown in bold.

| Locus | Length | Gene function | No. of variable sites | SNV density |
| --- | --- | --- | --- | --- |
| **TP_0136** | **1488** | **Fibronectin-binding protein** | **68** | **0.045698925** |
| **TP_0462** | **1179** | **Putative lipoprotein** | **43** | **0.036471586** |
| **TP_0548** | **1305** | **Rare outer membrane protein,**  **FadL** | **38** | **0.029118774** |
| TP_0858 | 1227 | Hypothetical protein | 34 | 0.027709861 |
| **TP_0865** | **1440** | **Putative outer membrane protein** | **30** | **0.020833333** |
| TP_0488 | 2538 | Methyl-accepting chemotaxis protein | 27 | 0.010638298 |
| TP_1019 | 444 | Aspartyl/Glutamyl tRNA | 4 | 0.009009009 |
| TP_0326 | 2562 | Outer membrane protein, BamA | 19 | 0.007416081 |
| TP_0968 | 1623 | Hypothetical protein | 11 | 0.006777572 |
| TP_0966 | 1635 | Hypothetical protein | 9 | 0.005504587 |
| TP_0558 | 909 | Putative nickel-cobalt transporter | 5 | 0.00550055 |
| TP_0515 | 2976 | Putative outer membrane protein | 16 | 0.005376344 |
| TP_0967 | 1554 | Hypothetical protein | 8 | 0.005148005 |
| TP_0179 | 1884 | Hypothetical protein | 9 | 0.00477707 |
| TP_0304 | 3120 | Hypothetical protein | 10 | 0.003205128 |
| **TP_0705** | **2655** | **Carboxypeptidase/penicillin-binding protein** | **5** | **0.001883239** |
| TP_0898 | 3720 | Exodeoxyribonuclease V beta subunit | 4 | 0.001075269 |

Please note, that the SNV density was only one (out of three) criteria for the selection of candidate loci.
