# Supplementary material for: Molecular characterization of Treponema pallidum subsp. pallidum in Switzerland and France with a new multilocus sequence typing scheme
Source: PLoS One. 2018 Jul 30;13(7):e0200773. doi: 10.1371/journal.pone.0200773 (PMC6066202; doi:10.1371/journal.pone.0200773)
Supplement: S3 Table — (DOCX) [file pone.0200773.s004.docx]

**Table S3. Primers used for the nested-PCR amplification for the candidate loci and the 23S rRNA gene.**

| Locus | External primers (5-3) | Coordinates^1^ | Length of PCR product | Source/reference | Internal primers (5-3) | Coordinates^1^ | Length of PCR product | Source |
| --- | --- | --- | --- | --- | --- | --- | --- | --- |
| TP0136 | AACCCGTTAGCGCCCAACAT | 157804-157823 | 1789 bp | [16] | AGTGTCTTCCTCGTCCGTTC | 158206-158225 | 1206 bp | [30] |
|  | TCCCAGCTCAGCCGAATCTC | 159570-159589 |  |  | CACGTGGTGGTGTCAAACTT | 159392-159411 |  |  |
| TP0462 | GTAGTCCAAGCAGGTGGAGG | 492548-492567 | 721 bp | This study | AGTCTCTTAACCGGCTGCGA | 492664-492683 | 528 bp | This study |
|  | AGGATGGGGGACGTAGTAGC | 493249-493268 |  |  | GTACACGGCCTTGGTGGTTA | 493172-493191 |  |  |
| TP0548 | TGGGGCACTAAACCGGAAGA | 593136-593155 | 1567 bp | [16] | GCGGTCCCTATGATATCGTGT | 593285-593305 | 1065 bp | [30] |
|  | TACGGGCATTTGCGGATAGG | 594683-594702 |  |  | GAGCCACTTCAGCCCTACTG | 594330-594349 |  |  |
| TP0705 | GGTCTATATGCAGCCCTTCTTC | 772663-772684 | 1181 bp | This study | TGCGGCTTATCCTGATGAATAG | 772917-772938 | 803 bp | This study |
|  | GCTTGAGAACGATACCGGATAC | 773822-773843 |  |  | TATTCTGCGGCGTTGGATAG | 773700-773719 |  |  |
| TP0865 | GTGCCAAAGAGAAACCACCG | 945439-945458 | 844 bp | This study | CGGCTCATGGGAACCAAAAT | 945608-945622 | 621 bp | This study |
|  | TGTGTCTTGCCCCGCTTTTT | 946263-946282 |  |  | AAGGGGGTGAAGTTCTGGGG | 946209-946228 |  |  |
| 23S rDNA^2^ | CGAAGGGAAGCAGGTGTAGT | 234704-234723, 283149-283168 | 1666 and 1658 bp | This study, [31] | GTACCGCAAACCGACACAG | 234768-234786 | 629 bp | [31] |
|  | GCGCGAACACCTCTTTTTAC | 236350-236369 |  |  | AGTCAAACCGCCCACCTAC | 235378-235396 |  |  |
|  | GAACCGTCCCTGAAAACTCA | 284787-284806 |  |  |  |  |  |  |

^1^According to the Nichols genome (CP004010.2).

^2^Both copies of 23S rDNA gene were amplified.

The primers were designed according to the lengths of the variable regions in order to optimize the discriminating ability.
