# Supplementary material for: Molecular characterization of Treponema pallidum subsp. pallidum in Switzerland and France with a new multilocus sequence typing scheme
Source: PLoS One. 2018 Jul 30;13(7):e0200773. doi: 10.1371/journal.pone.0200773 (PMC6066202; doi:10.1371/journal.pone.0200773)
Supplement: S4 Table — (DOCX) [file pone.0200773.s005.docx]

| GenBank accession number | Allelic variant |
| --- | --- |
| MG894098 | TP_0136_1 |
| MG894099 | TP_0136_2 |
| MG894100 | TP_0136_3 |
| MG894101 | TP_0136_4 |
| MG894102 | TP_0136_5 |
| MG894103 | TP_0136_6 |
| MG894104 | TP_0136_7 |
| MG894105 | TP_0136_8 |
| MG894106 | TP_0136_9 |
| MG894107 | TP_0136_10 |
| MG894108 | TP_0136_12 |
| MG894109 | TP_0136_13 |
| MG894082 | TP_0548_1 |
| MG894083 | TP_0548_2 |
| MG894084 | TP_0548_3 |
| MG894085 | TP_0548_4 |
| MG894086 | TP_0548_5 |
| MG894087 | TP_0548_6 |
| MG894088 | TP_0548_7 |
| MG894089 | TP_0548_8 |
| MG894090 | TP_0548_9 |
| MG894091 | TP_0548_10 |
| MG894092 | TP_0548_11 |
| MG894093 | TP_0548_12 |
| MG894094 | TP_0548_13 |
| MG894095 | TP_0548_14 |
| MG894096 | TP_0548_15 |
| MG894097 | TP_0548_16 |
| MG894110 | TP_0705_1 |
| MG894111 | TP_0705_2 |
| MG894112 | TP_0705_3 |
| MG894113 | TP_0705_4 |
| MG894114 | TP_0705_5 |
| MG894115 | TP_0705_6 |
| MG894116 | TP_0705_7 |
| MG894117 | TP_0705_8 |
| MG894118 | TP_0705_9 |
| MG894119 | TP_0705_10 |

**Table S4: GenBank accession numbers corresponding to the particular allelic variants.**
