# Supplementary material for: Molecular characterization of Treponema pallidum subsp. pallidum in Switzerland and France with a new multilocus sequence typing scheme
Source: PLoS One. 2018 Jul 30;13(7):e0200773. doi: 10.1371/journal.pone.0200773 (PMC6066202; doi:10.1371/journal.pone.0200773)
Supplement: S5 Table — (DOCX) [file pone.0200773.s006.docx]

**Table S5.** **Nineteen different genotypes found among 99 typeable Swiss and French clinical samples by Sequencing-based molecular typing (SBMT).**

| Genotype^1^ | Typing | TP0136^2^ | TP0548^2^ | 23S rRNA gene | Genetic group |
| --- | --- | --- | --- | --- | --- |
| SSS | Complete | Identical to SS14 | Identical to SS14 | Sensitive | SS14-clade |
| SSR8 | Complete | Identical to SS14 | Identical to SS14 | A2058G | SS14-clade |
| SSR9 | Complete | Identical to SS14 | Identical to SS14 | A2059G | SS14-clade |
| SU2R8 | Complete | Identical to SS14 | Unique 2 | A2058G | SS14-clade |
| SU2S | Complete | Identical to SS14 | Unique 2 | Sensitive | SS14-clade |
| SU15R8^3^ | Complete | Identical to SS14 | Unique 15 | A2058G | SS14-clade |
| SU19R8^3^ | Complete | Identical to SS14 | Unique 19 | A2058G | SS14-clade |
| U5SS^3^ | Complete | Unique 5 | Identical to SS14 | Sensitive | SS14-clade |
| U9U2S^3^ | Complete | Unique 9 | Unique 2 | Sensitive | SS14-clade |
| U6U14R8^3^ | Complete | Unique 6 | Unique 14 | A2058G | Nichols-clade |
| SU16R8^3^ | Complete | Identical to SS14 | Unique 16 | A2058G | SS14-clade |
| XU17X^3^ | Partial | NA^4^ | Unique 17 | NA^4^ | Nichols-clade |
| SU18R8^3^ | Complete | Identical to SS14 | Unique 18 | A2058G | SS14-clade |
| XU20R8^3^ | Partial | NA^4^ | Unique 20 | A2058G | Nichols-clade |
| SU21X^3^ | Partial | Identical to SS14 | Unique 21 | NA^4^ | SS14-clade |
| U7U2X^3^ | Partial | Unique 7 | Unique 2 | NA^4^ | SS14-clade |
| U8U2X^3^ | Partial | Unique 8 | NA^4^ | NA^4^ | SS14-clade |
| XU6S | Partial | NA^4^ | Unique 6 | Sensitive | Nichols-clade |
| U3XX | Partial | Unique 3 | NA^4^ | NA^4^ | Nichols-clade |

^1^Genotypes were denoted using SBMT by a three-letter code [21]. Briefly, the first letter stands for the TP0136 sequence (S, identical to SS14; U, unique sequence compared to SS14 strain; X, not determined), the second letter stands for the TP0548 sequence (S, identical to SS14; U, unique sequence compared to SS14 strain; X, not determined), and the third letter stands for sensitivity/resistance to macrolide antibiotics (S, sensitive; R8, A2058G, resistant; R9, A2059G, resistant).

^2^SNVs which determinate the allele variants are shown in Figure 1B.

^3^New genotypes detected in this study.

^4^NA, not available.

X, allelic variant was not determined.
