# Supplementary material for: Molecular characterization of Treponema pallidum subsp. pallidum in Switzerland and France with a new multilocus sequence typing scheme
Source: PLoS One. 2018 Jul 30;13(7):e0200773. doi: 10.1371/journal.pone.0200773 (PMC6066202; doi:10.1371/journal.pone.0200773)
Supplement: S6 Table — (DOCX) [file pone.0200773.s007.docx]

**Table S6.** **Fifteen different subtypes found among 89 typeable Swiss and French clinical samples by ECDCT.^1^**

| Subtype^2^ | Typing | No. of *arp* repetitions | *tpr*EGJ | 83 bp long sequence of TP0548 |
| --- | --- | --- | --- | --- |
|  |  |  |  |  |
| X d/a | Partial | X | d | a |
| 14 d/f | Complete | 14 | d | f |
| 14 d/g | Complete | 14 | d | g |
| X d/z^3^ | Partial | X | d | z |
| 14 e/g | Complete | 14 | e | g |
| 15 d/f | Complete | 15 | d | f |
| 15 d/g | Complete | 15 | d | g |
| 7 d/f | Complete | 7 | d | f |
| 8 d/g | Complete | 8 | d | g |
| 13 d/g | Complete | 13 | d | g |
| X b/g | Partial | X | b | g |
| X m/X | Partial | X | m | X |
| X d/c | Partial | X | d | c |
| X j/X | Partial | X | j | X |
| XX/y^3^ | Partial | X | X | y |

^1^Due to the low volume of the DNA extract of clinical samples, we performed the ECDCT only on a subset of the clinical samples (n=96, instead of 120).

^2^Subtypes were denoted using ECDCT [11, 13]. Briefly, the number stands for number of repetitions in the *arp* gene, the following letter stands for RFLP type of *tprE*, *G* and *J* genes and the last letter stands for the 83-bp long ECDCT_TP0548 sequence.

^3^New genotypes detected in this study.

Subtype 14d/g was further divided into five allelic profiles with MLST (1.3.1, 4.3.1, 6.3.1, 1.3.7, 1.8.1), on the other hand, the MLST allelic profiles 1.1.1 and 1.3.1 were divided into two (14d/f and 7d/f) and four (14d/g, 14e/g, 8d/g and 15d/g) different ECDCT subtypes, respectively.

X, undetermined.
