# Supplementary figures and images for: Molecular characterization of Treponema pallidum subsp. pallidum in Switzerland and France with a new multilocus sequence typing scheme
Source: PLoS One. 2018 Jul 30;13(7):e0200773. doi: 10.1371/journal.pone.0200773 (PMC6066202; doi:10.1371/journal.pone.0200773)

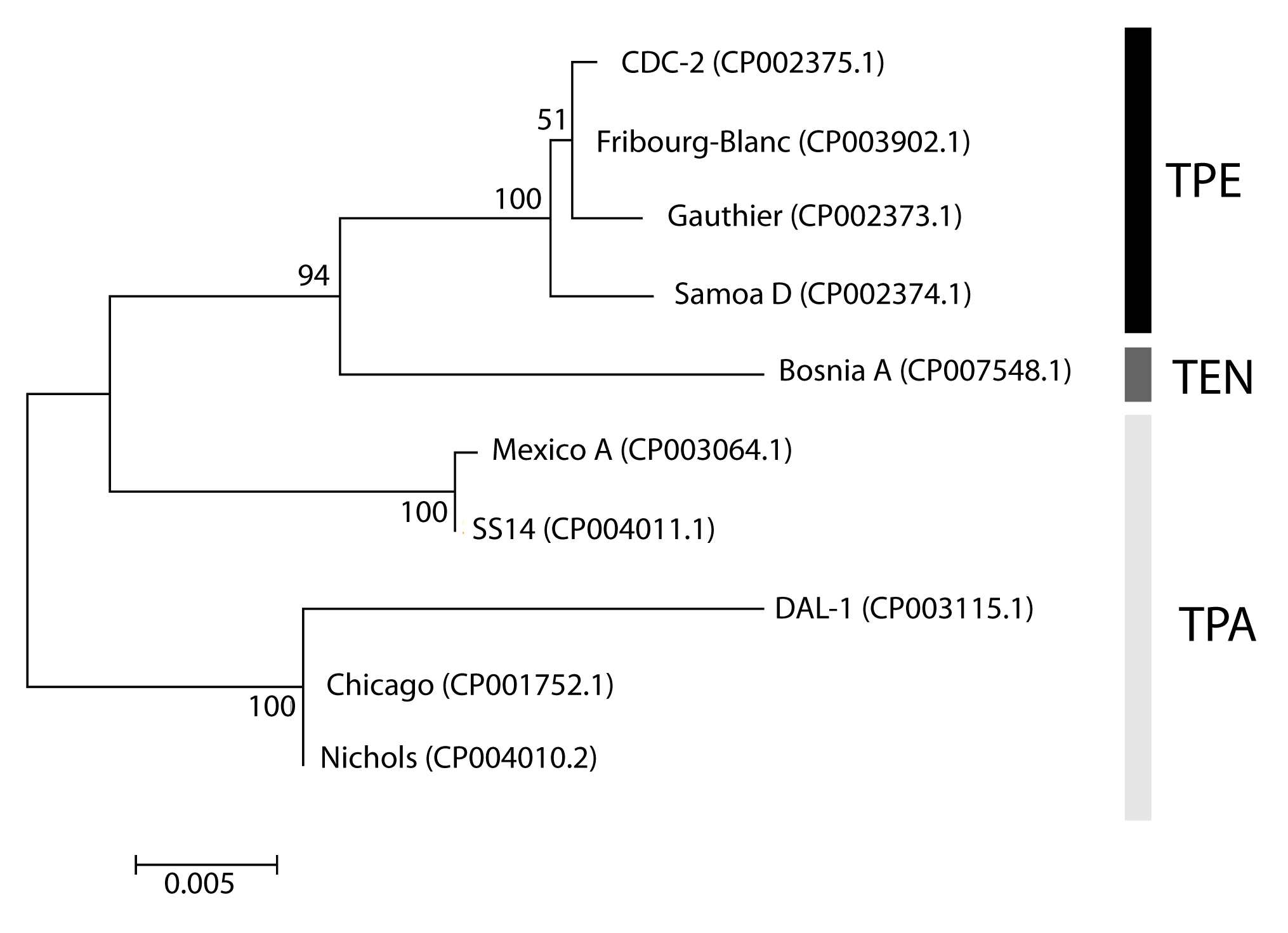

Supplement: S1 Fig — Maximum likelihood tree produced in MEGA 6 for concatenated sequences of typing loci (TP_0136, TP_0548, TP_0705) in available complete genomes of reference strains representing different Treponema pallidum subspecies (TPA–Treponema pallidum subsp. pallidum; TPE—Treponema pallidum subsp. pertenue; TEN—Treponema pallidum subsp. endemicum). (TIF) [file pone.0200773.s008.tif]
